# Optimization of Culture Conditions (Sucrose, pH, and Photoperiod) for *In Vitro* Regeneration and Early Detection of Somaclonal Variation in Ginger Lime (*Citrus assamensis*)

**DOI:** 10.1155/2014/262710

**Published:** 2014-04-01

**Authors:** Jamilah Syafawati Yaacob, Noraini Mahmad, Rosna Mat Taha, Normadiha Mohamed, Anis Idayu Mad Yussof, Azani Saleh

**Affiliations:** ^1^Institute of Biological Sciences, Faculty of Science, University of Malaya, 50603 Kuala Lumpur, Malaysia; ^2^Centre for Research in Biotechnology for Agriculture, Institute of Biological Sciences, Faculty of Science, University of Malaya, 50603 Kuala Lumpur, Malaysia; ^3^Faculty of Applied Science, MARA University of Technology, 40450 Shah Alam, Selangor, Malaysia

## Abstract

Various explants (stem, leaf, and root) of* Citrus assamensis* were cultured on MS media supplemented with various combinations and concentrations (0.5–2.0 mgL^−1^) of NAA and BAP. Optimum shoot and root regeneration were obtained from stem cultures supplemented with 1.5 mgL^−1^ NAA and 2.0 mgL^−1^ BAP, respectively. Explant type affects the success of tissue culture of this species, whereby stem explants were observed to be the most responsive. Addition of 30 gL^−1^ sucrose and pH of 5.8 was most optimum for * in vitro* regeneration of this species. Photoperiod of 16 hours of light and 8 hours of darkness was most optimum for shoot regeneration, but photoperiod of 24 hours of darkness was beneficial for production of callus. The morphology (macro and micro) and anatomy of * in vivo *and * in vitro*/*ex vitro Citrus assamensis *were also observed to elucidate any irregularities (or somaclonal variation) that may arise due to tissue culture protocols. Several minor micromorphological and anatomical differences were observed, possibly due to stress of tissue culture, but * in vitro* plantlets are expected to revert back to normal phenotype following full adaptation to the natural environment.

## 1. Introduction

*Citrus assamensis *is a member of Rutaceae, an important underexploited, hybrid, evergreen, and aromatic tree.* Citrus* sp. is very attractive due to their distinctive fruits, colours, and attractive smell, unique from other plants. Containing high amounts of vitamin C, citrus fruits can be consumed raw or extracted for production of highly nutritious beverages. The percentage of citrus juice is an extremely important parameter for its industrial processing, being also related to fruit size [1]. Furthermore,* Citrus *sp. can also be used as traditional medicine, whereby the smell of citrus leaves and fruits can overcome headache and nausea. Tissue culture of* Citrus* sp. is very important to increase the production and mass propagation of this valuable plant. The use of tissue culture in the breeding of* Citrus* sp. is essential as it has the potential to overcome infertility of citrus seeds due to fungal infections by* Fusarium, Rhizoctonia,* and* Sclerotium*. Fungal infections caused the citrus seeds to be damaged before they can be germinated [2].* Citrus* is generally propagated through budding, cutting, or layering. Therefore, propagation is limited to the period when buds are available [3].

*In vitro* micropropagation technology can overcome some constraints to* Citrus* improvement and cultivation and can increase fruit quality and resistance to disease and environmental stresses [4]. The size of the fruit is important not only because it is a component of productive yield but also because it determines the acceptance and demand by the consumers. The application of plant growth regulators can reenforce hormone balance in the peel, reducing or retarding precocious fall and unwelcome losses at harvest [5]. Very few literatures were found on* in vitro* regeneration of* Citrus* sp., particularly on* Citrus assamensis*. To the best of our knowledge, this is the first report describing the effects of different plant growth regulators on micropropagation of this species. The current study aimed to determine the optimum hormone for efficient regeneration of* Citrus assamensis *and study the effect of sucrose concentration, pH of the media, and photoperiod on organogenesis and callogenesis of this species. Ultimately, SEM and some histological studies were carried out on* in vitro* regenerants and compared with intact plants to detect early occurrence of somaclonal variation.

## 2. Materials and Methods

### 2.1. Effects of Different Concentrations and Combinations of NAA and BAP on Callogenesis and Organogenesis of* Citrus assamensis*

Seeds of* Citrus assamensis* obtained from “Rimba Ilmu” or botanical garden of University of Malaya, Malaysia, were first washed with nondiluted teepol, followed by tap water. The seeds were then sterilised with 99% (v/v) sodium hypochlorite solution for 1 min and rinsed three times with distilled water. In a laminar flow chamber, the seeds were dipped in 70% (v/v) ethanol for 1 min, blotted with sterile tissue paper, and cultured on solid MS basal media [6] to produce aseptic seedlings. The media were added with 30 gL^−1^ sucrose and 8 gL^−1^ agar. After four weeks, the stem, root, and leaf explants were cut into small pieces (3 mm^2^) and cultured on MS media with 22 different combinations and concentrations of *α*-naphthaleneacetic acid (NAA) and 6-benzyl aminopurine (BAP).

Each treatment was conducted in thirty replicates. The pH of the media was adjusted to 5.8 and autoclaved at 104 kPa (15 Psi^2^) and 121°C for 21 minutes. All cultures were maintained in a culture room at 25 ± 1°C, with a 16-hour photoperiod with 1.496 W m^−2^ of light intensity. Subcultures were performed every 21–28 days to provide new and fresh nutrients under the same conditions.

### 2.2. Effects of Different Concentrations of Sucrose, Photoperiod, and pH of Media on Callogenesis and Organogenesis of* Citrus assamensis*

Different explants of* Citrus assamensis* were transferred onto solid MS media supplemented with optimum hormone combination (1.5 mgL^−1^ NAA and 2.0 mgL^−1^ BAP) but with varied sucrose concentrations (10 gL^−1^, 20 gL^−1^, 30 gL^−1^, 40 gL^−1^, or 50 gL^−1^) and pH (4.8, 5.8, 6.8, 7.8, or 8.8). Cultures were maintained at 25 ± 1°C with 16 hours of light and 8 hours of darkness. Both experiments were conducted in thirty replicates. The effect of both sucrose concentrations and pH of the media on tissue culture of* Citrus assamensis* was monitored.

To study the effect of photoperiod on* in vitro* regeneration of* Citrus assamensis*, different explants of this species were cultured on MS media supplemented with 1.5 mgL^−1^ NAA and 2.0 mgL^−1^ BAP, 30 gL^−1^ sucrose, at pH 5.8. The cultures however were maintained under different light or photoperiod conditions, such as under 24 hours of darkness, 8 hours of light and 16 hours of darkness, 12 hours of light and 12 hours of darkness, 16 hours of light and 8 hours of darkness, and 24 hours of light.

### 2.3. Morphology and Anatomy of* In Vivo* and* Ex Vitro Citrus assamensis* Plants

Scanning electron microscope (SEM) was used to observe the differences between* in vivo *(intact) and* in vitro *leaves of* Citrus assamensis*. Number of stomata and trichomes present on adaxial and abaxial surfaces of the leaves were compared. Standard method and procedure for preparation of samples for scanning electron microscopy as described by Islam et al. [7] were followed.

### 2.4. Statistical Analysis

Randomized complete block design (RCBD) was employed in all experiment. Data analysis was conducted through analysis of variance and Duncan's multiple range test (DMRT) at 5% significance level.

## 3. Results and Discussion

### 3.1. In Vitro Regeneration of* Citrus assamensis*

**Figure 1:**
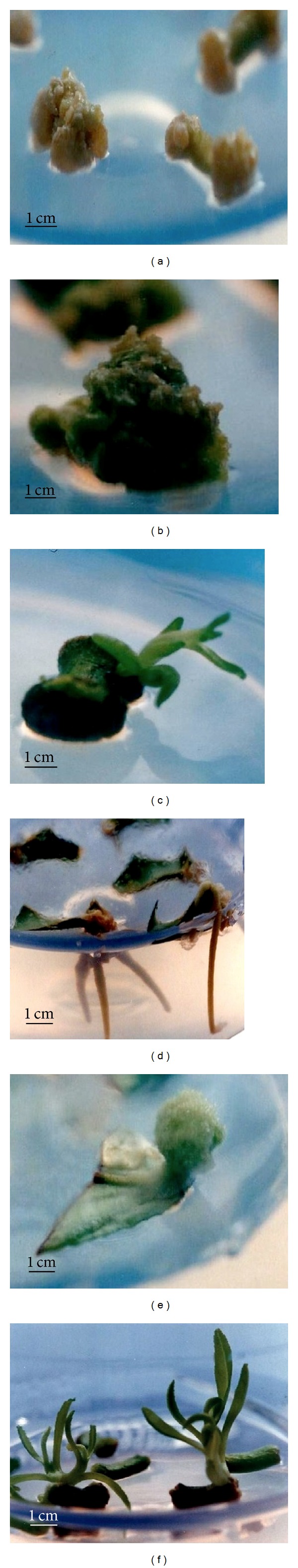
Various explants cultured on optimum regeneration media (MS media supplemented with 1.5 mgL^−1^ NAA and 2.0 mgL^−1^ BAP), maintained under 16 hours of light and 8 hours of darkness, with light intensity of 1000 lux. (a) Formation of yellow callus from root explant, (b) formation of green callus from root explant, (c) formation of green callus and production of shoot primordia from stem explant, (d) formation of roots from leaf explant after 18 days of culture, (e) formation of green callus from leaf explant, and (f) formation of multiple shoots from stem explant.

In general, all explant cultures (stem, root, or leaf) had the potential to generate callus although stem and root explants were found to be more responsive than leaf explants. The highest callus formation was produced from cultures supplemented with 1.5 mgL^−1^ NAA and 0.5 mgL^−1^ BAP, whereby 100%, 60%, and 20% of stem, root, and leaf explants had produced callus, respectively (Tables 1, 2, and 3). MS media added with other hormone combinations were also found to yield production of callus, such as 1.0 mgL^−1^ NAA and 0.5 mgL^−1^ BAP as well as 2.0 mgL^−1^ NAA and 2.0 mgL^−1^ BAP (Tables 1–3). Stem explants were the most responsive for callus induction, yielding light green callus after only 20 days of culture (Figure 1). Green callus was also produced from leaf cultures after 40 to 50 days (Figure 1) although the explant type was the least responsive. However, root explants were found to yield a mixture of yellow (Figure 1) and green callus. Comparable results were obtained by Mukhri and Yamaguchi [8], whereby formation of callus was reported from* Curcuma domestica* rhizomes when the regeneration media were supplemented with BAP and 2,4-D combined as well as BAP and NAA combined. On the other hand, organogenesis was yielded when* C. domestica* rhizomes were cultured on Ringe and Nitsch [9] medium added with only BAP.

Direct regeneration of shoots was produced from stem and root cultures, while leaf cultures showed limited organogenesis potential. The highest percentage of shoot formation (46.7%) was achieved when stem explants were cultured on MS media supplemented with 1.5 mgL^−1^ NAA and 2.0 mgL^−1^ BAP (Figure 1). It was also observed that high cytokinin (BAP) levels (2.0 mgL^−1^) aided the formation of shoots from explant cultures, compared to when low levels of cytokinin (1.0 mgL^−1^ BAP) were used. Formation of roots occurred readily from stem, root, and leaf explants cultured on MS media supplemented with 1.0 mgL^−1^ and 2.0 mgL^−1^ NAA. It was observed that root explants were the most responsive, yielding formation of roots within 18 days of culture compared to other explant types, which showed rooting after approximately 30 days. Furthermore, it was found that rooting occurred faster (18 days) when high auxin (NAA) concentrations (2.0 mgL^−1^) were used compared to 30 days, when low NAA concentrations (1.0 mgL^−1^) were used. Induction of rooting from leaf explant and production of multiple shoots from stem explant were depicted in Figure 1.

In general, tissue culture technology can be applied onto all plant species due to the basis that all plant parts can be utilized as explants. However, previous research suggested that herbaceous plants are generally more responsive and easily propagated via tissue culture protocols compared to woody plants. According to Abbot [10], woody plants exhibited lower propagation potential due to their complex and long life cycle. Clonal multiplication from mature tissues or organs of woody plants was difficult [11, 12]. Robb [13] stated that shoots of woody plants had long dormancy time than herbaceous plants; hence limited reports were found on successful* in vitro* propagation of woody plants.

Induction of callus often occurs at the wound site or the area from which the explant was cut, as shown by callus production from* Cucumis sativus*,* Cucumis melo,* and* Cucumis metuliferus* [14]. NAA and BAP when used in combination would encourage cell division [15]. Upadhyang et al. [16] also reported similar observations in* Rauvolfia caffra*, where 2.0 mgL^−1^ NAA and 2.0 mgL^−1^ BAP combined were most effective for production of callus. The use of auxin NAA was also beneficial in induction of rooting in tissue culture [14]. Induction of roots from shoot explants in woody plants occurred faster in growth media supplemented with IAA compared to when NAA was used [17].

The effect of sucrose concentrations, pH of the media, and photoperiod on tissue culture of* Citrus hybrids* was also studied. It was found that shoot formation was optimum when the media were supplemented with 30 gL^−1^ and 40 gL^−1^ sucrose (Table 4). Production of callus was the highest in stem and root cultures supplemented with 50 gL^−1^ sucrose (Table 4). However, it was observed that shoot and callus formation occurred faster (25 to 30 days) in media added with 30 gL^−1^ sucrose compared to media containing 20 gL^−1^ and 50 gL^−1^ sucrose (40 to 50 days).

Furthermore, pH lower or higher than 5.8 was also found to affect formation of shoots from explant cultures. It was observed that explant cultures with pH of the growth media adjusted to 5.8, 4.8, and 6.8 yielded percentage of shoot formation of 46.7%, 33.3%, and 20.0%, respectively (Table 5). Shoot formation also occurred faster (30 days) in media at pH 5.8 compared to 45 to 50 days in media at pH 4.8 and 6.8 (Table 5). However, root cultures in media at pH 4.8 showed significantly poorer formation of callus than in media at pH 5.8 and 6.8. Although similar percentage of callus formation (46.7%) was recorded from root cultures in media at pH 5.8 and 6.8, induction of callus was found to occur faster (30 days) at pH 5.8 than at pH 6.8 (35 days). Owen et al. [18] stated that pH of the media in tissue culture system can influence* in vitro shoot* multiplication, floral and secondary metabolites development, organogenesis, production of adventitious roots, and cell division.

Photoperiod also affects formation of shoots, multiple shoots, and callus. Percentage of shoot formation was found to increase when duration of exposure to light was increased, whereby optimum shoot formation (46.7%) was observed when the stem cultures were maintained under 16 hours of light and 8 hours of darkness (Table 6). However, exposure to 24 hours of darkness had reduced the percentage of shoot formation to 33.3% (Table 6). The lack of light also aided callus formation, whereby stem cultures exhibited percentage of callus formation of 73.3% when stem cultures were maintained under 24 hours of darkness (Table 6).

### 3.2. Morphology and Anatomy of* In Vivo* and* In Vitro* Grown* Citrus assamensis* Plants

The morphology of 3-month-old* in vivo *and acclimatized* in vitro *grown* Citrus assamensis *plantlets was observed and compared to detect any morphological irregularities that might have occurred due to tissue culture protocols.* In vivo* plant was grown on soil and subjected to natural environment in the garden, while* in vitro* plantlets were taken out from the culture vessels and acclimatized in the greenhouse for 1 month prior to SEM and histological analysis. The mean plant height, leaf structure, leaf shape, and leaf diameter were measured and compared. Furthermore, leaf segments of* Citrus assamensis* grown* in vivo* and* in vitro* were also viewed under scanning electron microscope (SEM) (Jeol JSM-6400) to elucidate any micromorphological differences that might present, hence detecting any occurrences of somaclonal variation.

**Figure 2:**
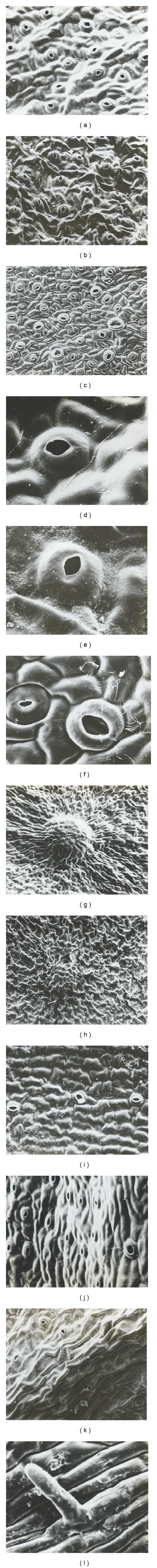
Scanning electron micrographs (SEM) of* in vivo* and* in vitro* grown* Citrus assamensis* leaves. (a) Abaxial surface of* in vitro* and (b)* in vivo* grown leaf showing anomocytic stomata; (c) abaxial surface of* in vitro* grown leaf, showing vivid anomocytic stomata in between lined epidermal cells; (d-e) abaxial surface of* in vivo* grown leaf showing clearly defined anomocytic stomata that appeared from the epidermal surface; (f) stoma of* in vitro* grown leaf sunk into epidermal surface; (g-h) abaxial surface of* in vivo* grown leaf, showing the presence of oil glands; (i) abaxial surface of* in vitro* grown leaf showing the absence of oil gland; (j-k) lack of trichomes was observed from primary veins of* in vivo *grown leaf; (l) primary vein of* in vitro* grown leaf showing the presence of trichome.

The microscopic studies of the structure of* in vivo *(intact) and* in vitro *leaves showed that the stomata apparatuses were generally anomocytic in shape. Oil glands were observed on both abaxial and adaxial surfaces of intact leaves but none on* in vitro* grown leaves (Figure 2). Stomata structures were observed on the abaxial surface of both* in vivo* and* in vitro* grown leaves but not on the adaxial surface (Figure 2). Interestingly, trichomes were only observed on primary veins of* in vitro* grown leaves (on abaxial surface) but none was present on* in vivo* grown leaves (Figure 2). SEM micrographs showing the stomata and trichome structures of* in vivo* and* in vitro* grown leaves of* Citrus assamensis* are depicted in Figure 2.

**Figure 3:**
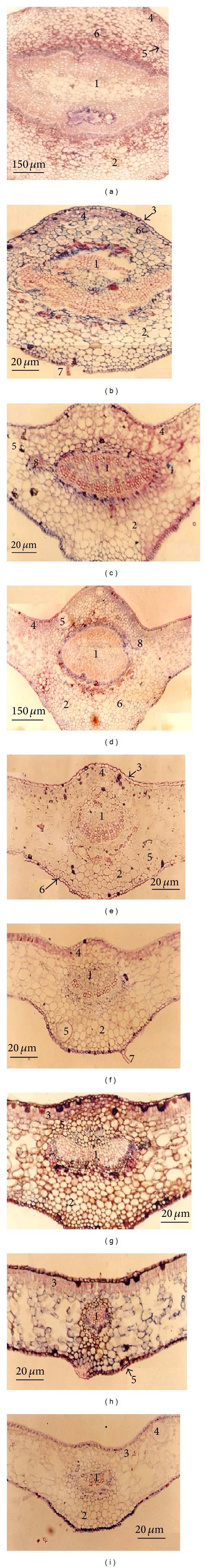
Histological examinations on* in vivo* and* in vitro* grown* Citrus assamensis* leaves. (a–c) Cross sections of leaf base of (a-b)* in vivo* and (c)* in vitro* plants, showing the presence of (1) vascular system, (2) cell structures, (3) cuticle, (4) palisade layer, (5) oil gland, (6) drusa, (7) trichome, and (8) lateral vascular bundle. (d–f) Cross sections of leaf midrib of (d-e)* in vivo* and (f)* in vitro* plants, showing the presence of (1) vascular system, (2) cell structures, (3) cuticle, (4) palisade layer, (5) oil gland, (6) drusa, (7) trichome, and (8) lateral vascular bundle. (g–i) Cross sections of leaf tip (apex) of (g-h)* in vivo* and (i)* in vitro* plants, showing the presence of (1) vascular system, (2) cell structures, (3) palisade layer, (4) oil gland, and (5) drusa.

Histological studies conducted on both* in vivo* and* in vitro* grown leaves revealed that* in vivo* leaf had “V” shaped vascular system, with uneven hexagonal, circular, and oval shaped cells (Figure 3). The cuticle was observed to be thin with three layers of palisade cells (Figure 3).* In vivo* grown* Citrus assamensis* leaf also lacked oil glands, although a lot of “drusa” were present (Figure 3). On the other hand,* in vitro* leaf had “elliptical” vascular system, also with uneven hexagonal-, circular-, and oval-shaped cells (Figure 3). However, no cuticle layer was observed on the* in vitro* leaf, although two vague palisade layers appeared to be present (Figure 3). In contrast with the* in vivo* leaf, oil gland was present on the* in vitro* leaf, but “drusa” was absent.

## 4. Conclusions

*Citrus assamensis *can be regenerated and propagated through tissue culture technique, as clearly demonstrated in the present investigation. Young stem was the most responsive explant type. The highest shoot formation (46.7%) was observed on MS medium supplemented with 1.5 mgL^−1^ NAA and 2.0 mgL^−1^ BAP. Acclimatization of* in vitro* plantlets of* Citrus assamensis *was successful and the plantlets had survived after 2 months of being transferred to greenhouse. This process is important to examine the success of this technique in propagation of* Citrus assamensis. *Meanwhile, xylem and phloem tissue in the leaf vascular bundle* in vivo* were thicker and more complex than in* in vitro* leaf vascular bundle, when compared anatomically. Furthermore, SEM showed more scattered and different shape of stoma* in vitro* compared to* in vivo. *However, most of* in vitro* stomata were submerged compared to* in vivo,* where the stomata structures were observed to arise from the epidermal surface. Based on SEM and histological studies, only minor micromorphological differences were observed, with no distinct indication of somaclonal variation.

## Figures and Tables

**Table 1:** The effects of different combinations and concentrations of BAP and NAA on stem explants of *Citrus hybrids*, cultured on solid MS media at 25 ± 1°C. Cultures were maintained at 16 hours of light and 8 hours of darkness, with 1000 lux intensity of light.

MS + hormone		Observations	Callus (%)	Organogenesis	
BAP (mgL^−1^)	NAA (mgL^−1^)			Shoot (%)	Root (%)
0.0	1.0	White callus formation after 20 days Root formation after 30 days	73.3 ± 0.6^ef^	NR	26.7 ± 0.6^a^
0.0	2.0	White callus formation after 15 days Root formation after 30 days	80.0 ± 5.8^fg^	NR	26.7 ± 1.7^a^
1.0	0.0	Shoot formation after 55 days	NR	20.0 ± 2.9^a^	NR
2.0	0.0	Shoot formation after 55 days	NR	20.0 ± 5.8^a^	NR
0.5	0.5	Green callus formation after 16 days Shoot formation after 60 days	80.0 ± 2.9^fg^	20.0 ± 1.2^a^	NR
1.0	0.5	Greenish callus formation after 30 days Shoot formation after 58 days	66.7 ± 1.2^de^	20.0 ± 2.9^a^	NR
1.5	0.5	Greenish callus formation after 16 days	53.3 ± 1.7^cd^	NR	NR
2.0	0.5	Greenish callus formation after 25 days Shoot formation after 58 days	60.0 ± 5.8^cde^	20.0 ± 2.3^a^	NR
0.5	1.0	Greenish callus formation after 20 days Shoot formation after 35 days	86.7 ± 2.3^gh^	20.0 ± 0.6^a^	NR
1.0	1.0	Greenish callus formation after 28 days	46.7 ± 0.6^bc^	NR	NR
1.5	1.0	Yellowish callus formation after 28 days Shoot formation after 50 days	26.7 ± 3.8^a^	20.0 ± 7.5^a^	NR
2.0	1.0	Greenish callus formation after 30 days	33.3 ± 5.7^ab^	NR	NR
0.5	1.5	Green callus formation after 20 days	100.0 ± 0.0^h^	NR	NR
1.0	1.5	Green callus formation after 30 days Shoot formation after 50 days	33.3 ± 2.3^ab^	20.0 ± 1.2^a^	NR
1.5	1.5	Greenish callus formation after 21 days	73.3 ± 2.3^efg^	NR	NR
2.0	1.5	Green callus formation after 25 days Shoot formation after 30 days	33.3 ± 1.2^ab^	46.7 ± 5.2^b^	NR
0.5	2.0	Green callus formation after 18 days Shoot formation after 40 days	73.3 ± 4.6^efg^	20.0 ± 2.9^a^	NR
1.0	2.0	Green callus formation after 20 days Shoot formation after 50 days	46.7 ± 4.0^bc^	20.0 ± 1.2^a^	NR
1.5	2.0	Yellow callus formation after 18 days Shoot formation after 55 days	60.0 ± 11.5^cde^	20.0 ± 3.5^a^	NR
2.0	2.0	Green callus formation after 18 days Shoot formation after 38 days	86.7 ± 5.8^gh^	26.7 ± 1.7^a^	NR
3.0	3.0	Yellowish callus formation after 28 days	46.7 ± 5.8^bc^	NR	NR

Mean ± SE, *n* = 30. Mean values with different letters in the same column are significantly different at *P* < 0.05 (NR: no response).

**Table 2:** The effects of different combinations and concentrations of BAP and NAA on root explants of *Citrus hybrids*, cultured on solid MS media at 25 ± 1°C. Cultures were maintained at 16 hours of light and 8 hours of darkness, with 1000 lux intensity of light.

MS + hormone		Observations	Callus (%)	Organogenesis	
BAP (mgL^−1^)	NAA (mgL^−1^)			Shoot (%)	Root (%)
0.0	1.0	White callus formation after 35 days Root formation after 35 days	46.7 ± 1.2^ab^	NR	20.0 ± 1.7^a^
0.0	2.0	White callus formation after 35 days Root formation after 35 days	40.0 ± 2.9^a^	NR	20.0 ± 3.5^a^
1.0	0.0	Shoot formation after 60 days	NR	20.0 ± 0.6^a^	NR
2.0	0.0	Shoot formation after 60 days	NR	20.0 ± 2.9^a^	NR
0.5	0.5	Yellowish callus formation after 30 days	93.3 ± 0.6^h^	NR	NR
1.0	0.5	Greenish callus formation after 30 days	93.3 ± 1.7^h^	NR	NR
1.5	0.5	Yellowish callus formation after 18 days	60.0 ± 1.6^cd^	NR	NR
2.0	0.5	Brown callus formation after 25 days	53.3 ± 4.0^bc^	NR	NR
0.5	1.0	Yellowish callus formation after 30 days	66.7 ± 1.2^de^	NR	NR
1.0	1.0	Yellowish callus formation after 22 days	40.0 ± 1.6^a^	NR	NR
1.5	1.0	Yellowish callus formation after 25 days	40.0 ± 6.4^a^	NR	NR
2.0	1.0	Yellow callus formation after 20 days	60.0 ± 1.2^cd^	NR	NR
0.5	1.5	Green callus formation after 25 days	60.0 ± 6.6^cd^	NR	NR
1.0	1.5	Brown callus formation after 25 days Shoot formation after 55 days	86.7 ± 1.8^gh^	20.0 ± 1.2^a^	NR
1.5	1.5	Yellow callus formation after 25 days	66.5 ± 3.5^de^	NR	NR
2.0	1.5	Yellow callus formation after 30 days Root formation after 50 days	46.7 ± 1.2^ab^	NR	20.0 ± 1.2^a^
0.5	2.0	Yellow callus formation after 16 days	46.7 ± 2.9^ab^	NR	NR
1.0	2.0	Greenish callus formation after 18 days Shoot formation after 55 days	80.0 ± 8.6^fg^	20.0 ± 4.6^a^	NR
1.5	2.0	Yellowish callus formation after 18 days	66.7 ± 1.7^de^	NR	NR
2.0	2.0	Yellowish callus formation after 16 days	73.3 ± 1.7^ef^	NR	NR
3.0	3.0	Yellow callus formation after 30 days	60.0 ± 3.2^cd^	NR	NR

Mean ± SE, *n* = 30. Mean values with different letters in the same column are significantly different at *P* < 0.05 (NR: no response).

**Table 3:** The effects of different combinations and concentrations of BAP and NAA on leaf explants of *Citrus hybrids*, cultured on solid MS media at 25 ± 1°C. Cultures were maintained at 16 hours of light and 8 hours of darkness, with 1000 lux intensity of light.

MS + hormone		Observations	Callus (%)	Organogenesis	
BAP (mgL^−1^)	NAA (mgL^−1^)			Shoot (%)	Root (%)
0.0	1.0	Root formation after 30 days	NR	NR	26.7 ± 2.3^a^
0.0	2.0	Root formation after 30 days	NR	NR	53.3 ± 1.2^b^
1.0	0.0	No response	NR	NR	NR
2.0	0.0	No response	NR	NR	NR
0.5	0.5	No response	NR	NR	NR
1.0	0.5	No response	NR	NR	NR
1.5	0.5	Green callus formation after 35 days	20.0 ± 1.2^a^	NR	NR
2.0	0.5	Green callus formation after 40 days	26.7 ± 2.9^a^	NR	NR
0.5	1.0	Green callus formation after 45 days	20.0 ± 1.7^a^	NR	NR
1.0	1.0	No response	NR	NR	NR
1.5	1.0	Yellowish callus formation after 50 days	20.0 ± 1.2^a^	NR	NR
2.0	1.0	No response	NR	NR	NR
0.5	1.5	Green callus formation after 38 days	20.0 ± 4.0^a^	NR	NR
1.0	1.5	Green callus formation after 30 days	20.0 ± 0.5^a^	NR	NR
1.5	1.5	No response	NR	NR	NR
2.0	1.5	No response	NR	NR	NR
0.5	2.0	No response	NR	NR	NR
1.0	2.0	Green callus formation after 50 days	20.0 ± 2.7^a^	NR	NR
1.5	2.0	Green callus formation after 40 days	20.0 ± 1.7^a^	NR	NR
2.0	2.0	Green callus formation after 40 days	26.7 ± 2.3^a^	NR	NR
3.0	3.0	No response	NR	NR	NR

Mean ± SE, *n* = 30. Mean values with different letters in the same column are significantly different at *P* < 0.05 (NR: no response).

**Table 4:** The effects of different concentrations of sucrose on stem, root, and leaf explants, cultured on optimum media (MS + 1.5 mgL^−1^ NAA + 2.0 mgL^−1^ BAP) at 25 ± 1°C. Cultures were maintained at 16 hours of light and 8 hours of darkness, with 1000 lux intensity of light.

Concentrations of sucrose (gL^−1^)	Explants	Observations	Callus (%)	Organogenesis	
				Shoot (%)	Root (%)
20	Stem	Shoots formation after 60 days	NR	26.7 ± 1.2^a^	NR
	Root	Green callus formation after 50 days	20.0 ± 1.7^a^	NR	NR
	Leaf	No response	NR	NR	NR

30	Stem	Green callus formation after 25 days Shoots formation after 30 days	33.3 ± 1.2^a^	46.7 ± 1.7^b^	NR
	Root	Yellow callus formation after 30 days	46.7 ± 1.7^c^	NR	NR
	Leaf	No response	NR	NR	NR

40	Stem	Shoots formation after 50 days	NR	30.0 ± 2.9^a^	NR
	Root	Green callus formation after 40 days	30.0 ± 3.5^b^	NR	NR
	Leaf	No response	NR	NR	NR

50	Stem	Green callus formation after 40 days Shoots formation after 50 days	66.7 ± 3.5^b^	26.7 ± 1.2^a^	NR
	Root	Yellow callus formation after 40 days	46.7 ± 2.0^c^	NR	NR
	Leaf	No response	NR	NR	NR

Mean ± SE, *n* = 30. Mean values with different letters (subject to different explant type) in the same column are significantly different at *P* < 0.05 (NR: no response).

**Table 5:** The effects of different pH on stem, root, and leaf explants, cultured on optimum media (MS + 1.5 mgL^−1^ NAA + 2.0 mgL^−1^ BAP) at 25 ± 1°C. Cultures were maintained at 16 hours of light and 8 hours of darkness, with 1000 lux intensity of light.

pH of media	Explants	Observations	Callus (%)	Organogenesis	
				Shoot (%)	Root (%)
4.8	Stem	Shoots formation after 45 days	NR	33.3 ± 1.7^b^	NR
	Root	Yellow callus formation after 35 days	33.3 ± 1.2^a^	NR	NR
	Leaf	No response	NR	NR	NR

5.8	Stem	Green callus formation after 25 days Shoots formation after 30 days	33.3 ± 2.3^a^	46.7 ± 2.3^c^	NR
	Root	Yellow callus formation after 30 days	46.7 ± 3.5^b^	NR	NR
	Leaf	No response	NR	NR	NR

6.8	Stem	Shoots formation after 55 days	NR	20.0 ± 2.3^a^	NR
	Root	Green callus formation after 50 days	46.7 ± 1.7^b^	NR	NR
	Leaf	No response	NR	NR	NR

Mean ± SE, *n* = 30. Mean values with different letters (subject to different explant type) in the same column are significantly different at *P* < 0.05 (NR: no response).

**Table 6:** The effects of light exposure on stem, root, and leaf explants, cultured on optimum media (MS + 1.5 mgL^−1^ NAA + 2.0 mgL^−1^ BAP) at 25 ± 1°C. Cultures were maintained at 16 hours of light and 8 hours of darkness, with 1000 lux intensity of light.

Light exposure (hrs)		Explant	Observations	Callus (%)	Organogenesis	
Light	Darkness				Shoot (%)	Root (%)
0	24	Stem	White callus formation after 12 days	73.3 ± 4.6^b^	NR	NR
		Root	White callus formation after 35 days	46.7 ± 2.3^a^	NR	NR
		Leaf	No response	NR	NR	NR

8	16	Stem	Multiple shoots formation after 16 days	NR	20.0 ± 1.7^a^	NR
		Root	Yellow callus formation after 40 days	40.0 ± 1.7^a^	NR	NR
		Leaf	No response	NR	NR	NR

12	12	Stem	Green callus formation after 45 days Shoots formation after 50 days	40.0 ± 1.7^a^	40.0 ± 2.3^bc^	NR
		Root	Green callus formation after 25 days	86.7 ± 1.2^c^	NR	NR
		Leaf	No response	NR	NR	NR

16	8	Stem	Green callus formation after 25 days Shoots formation after 50 days	33.3 ± 2.3^a^	46.7 ± 5.2^c^	NR
		Root	Yellow callus formation after 30 days	46.7 ± 3.5^a^	NR	NR
		Leaf	No response	NR	NR	NR

24	0	Stem	Multiple shoots formation after 14 days	NR	33.3 ± 1.7^b^	NR
		Root	Green yellow formation after 30 days	60.0 ± 2.3^b^	NR	NR
		Leaf	No response	NR	NR	NR

Mean ± SE, *n* = 30. Mean values with different letters (subject to different explant type) in the same column are significantly different at *P* < 0.05 (NR: no response).
